# Mice lacking galectin-3 (*Lgals3*) function have decreased home cage movement

**DOI:** 10.1186/s12868-018-0428-x

**Published:** 2018-05-02

**Authors:** Tammy R. Chaudoin, Stephen J. Bonasera

**Affiliations:** Division of Geriatrics, Department of Internal Medicine, University of Nebraska Medical Center, 3028 Durham Research Center II, Omaha, NE 68198-5039 USA

**Keywords:** Circadian rhythm, Galectin-3, Ingestive behavior, *Lgals3*, Locomotor behavior

## Abstract

**Background:**

Galectins are a large family of proteins evolved to recognize specific carbohydrate moieties. Given the importance of pattern recognition processes for multiple biological tasks, including CNS development and immune recognition, we examined the home cage behavioral phenotype of mice lacking galectin-3 (*Lgals3*) function. Using a sophisticated monitoring apparatus capable of examining feeding, drinking, and movement at millisecond temporal and 0.5 cm spatial resolutions, we observed daily behavioral patterns from 10 wildtype male C57BL/6J and 10 *Lgals3* constitutive knockout (*Lgals3*^−/−^; both cohorts aged 2–3 months) mice over 17 consecutive days. We performed a second behavioral assessment of this cohort at age 6–7 months.

**Results:**

At both ages, *Lgals3*^−/−^ mice demonstrated less movement compared to wildtype controls. Both forward locomotion and movement-in-place behaviors were decreased in *Lgals3*^−/−^ mice, due to decreased bout numbers, initiation rates, and durations. We additionally noted perturbation of behavioral circadian rhythms in *Lgals3*^−/−^ mice, with mice at both ages demonstrating greater variability in day-to-day performance of feeding, drinking, and movement (as assessed by Lomb-Scargle analysis) compared to wildtype.

**Conclusion:**

Carbohydrate recognition tasks performed by *Lgals3* may be required for appropriate development of CNS structures involved in the generation and control of locomotor behavior.

**Electronic supplementary material:**

The online version of this article (10.1186/s12868-018-0428-x) contains supplementary material, which is available to authorized users.

## Background

Galectins are an evolutionarily ancient family of proteins sharing a high binding affinity for carbohydrates with β-galactoside linkages. In the extracellular space, galectins interact (through a conserved carbohydrate recognition domain, *aka* CRD) with glycosylated proteins to mediate both cell-to-cell interactions and cell-to-matrix adhesion. Galectins are thus pattern recognition molecules specialized to distinguish carbohydrate moieties.

Within the galectin family, galectin-3 (also known as *Lgals3*) has unique properties. Its preferred ligand is N-acetyllactosamine [1]. It is also the only galectin containing a conserved N-domain as well as a single CRD domain. This N-domain allows *Lgals3* not bound to a carbohydrate target to form multimeric complexes [2]. In this manner, low extracellular *Lgals3* concentrations tend to inhibit extracellular interactions and adhesion [3], while high *Lgals3* extracellular concentrations facilitate cellular adhesion [4, 5]. *Lgals3* affinity for ECM substrates is also modulated by phosphorylation at its Ser6 residue [6].

*Lgals3* is an NFκB target gene [7]; *Lgals3* protein is widely distributed throughout most tissue sites (as demonstrated by the TiGER Tissue specific gene expression and regulation database; [8]). Furthermore, within specific tissues, *Lgals3* protein expression is widespread, with extracellular [9], membrane bound, cytoplasmic, and nuclear localizations (for review, see [10]).

Given these varied *Lgals3* tissue and cellular distributions, it is not surprising to find that cellular functions attributed to *Lgals3* are numerous and diverse: (1) context-sensitive cell adhesion [11] or dehiscence [12], (2) receptor for advanced glycation (AGE) and advanced lipoxygenation (ALE) end products [13], (3) regulating clathrin-independent endocytosis [14], (4) regulating intracellular signal transduction by spacing apart membrane-bound signaling complexes [15], (5) modulating Wnt/β-catenin signaling [16], (6) influencing TTF-1 and STAT transcription factor activities [17, 18], (6) regulating mRNA maturation through their effects on spliceosome function [19], (7) repairing DNA damage [20], (8) inducing late G_1_ cell cycle arrest [21], (9) promoting cell proliferation [22], and (10) promoting cell survival through the anti-apoptotic effects of Bcl2 [23]. *Lgals3* also participates in immune function. It contributes to innate immunity through its abilities to opsonize cellular debris [24], facilitate generation of respiratory burst enzymes [25], function as a MerTK-specific eat-me signal [26], and act as a CNS alarmin [27]; moreover, it contributes to acquired immunity through its regulation of T cell activation [15].

Finally, there is an increasing recognition that proteins capable of molecular pattern recognition play significant roles in CNS synapse formation, pruning, and maintenance [28]. Already, many different classes of pattern recognition molecules have been implicated in these processes: major histocompatibility genes [29, 30], complement [31], paired immunoglobulin-like receptors [32], and toll-like receptors [33]. These pattern recognition receptors all have highly conserved glycosylation sites (MHC-I [34]; C3 [35]; PirB [36]; Tlr2 [37]), making them potential *Lgals3* interaction partners. *Lgals3*-mediated recognition of specific N-acetyllactosamine sites may be required for CNS developmental events. For example, prior studies demonstrate that altered *Lgals3* expression has a role in age-related synaptic changes accompanying functional loss [38].

### Objectives

We thus assessed baseline behaviors in a mouse model to evaluate *Lgals3* influence on important behaviors of clinical interest, including metabolism, feeding, drinking, movement, and circadian rhythm. We measured metabolism using indirect calorimetry with correction for mouse adiposity. We used a sophisticated home cage monitoring approach to assess mouse feeding, drinking, activity, and circadian rhythm in a noninvasive manner over more than 2 weeks of observation. Surprisingly, despite the large number of molecular interactions involving *Lgals3*, and well-documented *Lgals3* CNS expression (in neurons, microglia, and astrocytes), we found only two significant behavioral deficits accompanying constitutive *Lgals3* loss: decreased locomotor movement, and diminished fidelity of circadian feeding, drinking, and movement patterns.

## Methods

### Ethical statement

All studies were performed in full concordance with both institutional and federal regulations regarding animal care and use; our research protocol was approved by the University of Nebraska Medical Center (UNMC) Institutional Animal Care and Use Committee (IACUC).

### Animal models and husbandry

We evaluated cohorts of C57BL/6J male mice (stock number 000664) and mice carrying a constitutive *Lgals3* mutation (*Lgals3*^−/−^; B6.Cg-*Lgals3*^*tm1Poi*^/J; stock number 006338), both obtained from Jackson Laboratories. Briefly, these mice were created through homologous recombination removing the native *Lgals3* exons II, III, and IV and replacing them with a neomycin resistance cassette. Homozygous mutant offspring derived from this targeted lesion expressed only the 3.4 kb predicted *Eco*RI fragment, and did not have the 6.4 kb WT fragment [39]. Mice obtained from Jackson have undergone 7 backcrosses to C57BL/6J from the original chimeric mouse. Upon initial receipt at our vivarium, mice were housed in a microisolator system (Lab Products, Seaford DE) at a density of ≤ 5 mice per cage. No mice were used for breeding purposes. The facility maintained a 12:12 circadian lighting schedule with lights on at 06:00 CST; vivarium temperatures ranged between 20 and 23 °C. All mice had ad libitum access to food (Envigo Teklad #7012), water, and environmental enrichment (Crinkle Paper Pouches, WF Fisher). Animal health was checked on a daily basis by UNMC Comparative Medicine staff. Mice remained in the vivarium for 14 days prior to start of testing. While mice were housed in the vivarium, cage bedding, food, and water were changed every 14 days. Mice were returned to the vivarium and kept singly-housed between the two longitudinal assessments. Following testing, mice were sacrificed by CO_2_ inhalation followed by cervical dislocation.

### Body mass composition

We performed dual X-ray absorptiometry (DEXA) imaging to measure mouse adiposity. We performed a longitudinal assessment of 10 WT and 10 *Lgals3*^−/−^ mice; we first tested mice at 2–3 months old, followed by repeat assessment at 6–7 months old. Investigators were not blinded to mouse genotype. Mice received DEXA imaging in a random manner. DEXA testing occurred between 10:00 and 16:00. Animals were lightly anesthetized with isoflurane at 1–3 vol%, and imaged with a Piximus I (Inside/Outside, Fitchburg WI). Before data acquisition, the system was calibrated by imaging a phantom with defined radiological characteristics. We used vendor supplied software (Piximus I, GE Lunar) to identify regions of interest (ROIs) encompassing the mouse chest/abdomen/pelvis for determination of bone mass density (BMD), bone mineral content (BMC), bone area (BArea), tissue area (TArea), ratio of soft tissue attenuation (R_ST_), total tissue mass (TTM), and percent adiposity (% fat). Data was analyzed by repeated measures analysis of variance (ANOVA, implemented in MATLAB 2011b) with genotype and mouse age as primary factors, and a genotype × age interaction.

### Indirect calorimetry to assess basal and activity-associated metabolism

We performed a longitudinal assessment of 10 WT and 10 *Lgals3*^−/−^ mice; we first tested mice at 2–3 months old, followed by repeat assessment at 6–7 months old. Investigators were not blinded to mouse genotype. Mice were assigned to calorimetry enclosures in a random manner. Animals were fasted overnight, then placed into 8 hermetically-sealed metabolic cages (Oxymax, Columbus Instruments). Mice were tested between 10:00 and 17:00; each measurement of gas tension required 2 min, so each animal had its metabolic parameters assessed every 16 min during the testing period. We used vendor supplied software (Oxymax for Windows 4.49) to determine maximum oxygen uptake (${\dot{\text{V}}}$O_2_), global oxygen delivery (DO_2_), oxygen output (O_2_out), maximum CO_2_ production, (${\dot{\text{V}}}$CO_2_), global CO_2_ removal (DCO_2_), CO_2_ output (CO_2_out), and heat generated. Basal metabolic rates were determined by averaging measurements obtained during the 3 epochs displaying the least activity (as measured by photobeam brackets spanning the length of the metabolic chamber); similarly, activity-associated metabolic rates were determined by averaging measurements obtained during the 3 epochs displaying the most activity. Metabolic parameters were then adjusted for mouse adiposity and mouse lean body mass using ANCOVA [40, 41]. Full description of our metabolic testing apparatus is provided in [42]. Mice were weighed using a Scout Pro SP401 (Ohaus, Parsippany NJ) before indirect calorimetry, home cage behavioral monitoring, and on a weekly basis between the two longitudinal assessments and following the last assessment.

### Home cage behavioral monitoring

Details describing our home cage behavioral monitoring system have been previously published [43, 44]. Briefly, we measure mouse feeding, drinking, and movement at high temporal and spatial precision in a custom-designed home cage over extended periods of time. Feeding is quantified by the number of times a mouse breaks a photobeam while accessing a food hopper (ms resolution). Drinking is quantified by a capactive lickometer (ms resolution). Movement is quantified by solving exact equations of torque measured at three load cells and knowing mouse body weight (ms temporal, 0.5 cm spatial resolution). Data undergoes rigorous automated quality control, followed by behavioral classification and analysis [43]. We performed a longitudinal assessment of 10 WT and 10 *Lgals3*^−/−^ mice; we first tested mice at 2–3 months old, followed by repeat assessment at 6–7 months old. Mice were randomly assigned to one of 64 home cage behavioral assessment arenas. Each arena contained a niche modeled to approximate dimensions of a mouse burrow to organize mouse resting location, and a nestlet (NES3600, Ancare) for nesting materials. Powdered chow and water were available to all mice ad libitum; behavioral testing room lighting schedule was 12:12 with lights on at 0600 h CST. Room temperature ranged between 20 and 23 °C; facility relative humidity ranged between 35 and 51%. The arena floor was layered with absorbent bedding (200 ml Teklad Sani-chips (Envigo, Huntington UK), 300 ml ALPHA-Dri^®^ + PLUS (Shepherd Specialty Papers, Watertown TN)). Mice were allowed to habituate to the home cage monitoring system for 5 days before start of data collection. Investigators were not blinded regarding mouse genotype. We then collected 17 consecutive days of data for each mouse (to ensure that we had at least 14 full days of data after quality control) at each longitudinal assessment time point.

### Mouse behavioral data quality control, classification, and analysis

We employed automated data quality control checks to identify outliers and epochs where there may be questions regarding data integrity (in particular, blocked photobeams and sipper tube leaks). These epochs constituted less than 1% of total data collected, and were removed from the dataset. Following data quality control, individual mouse feeding, drinking, and movement events were classified to determine active and inactive state properties (in a manner similar to human actigraphy), and to fit these behaviors to a Gaussian mixture model that would allow us to compare bout structures across genotypes. Theory and implementation of these processes has been published in [42, 43 (both manuscript and data supplement), 44].

Following data classification, we performed false-discovery rate (FDR) analysis over 665 different outcomes that assessed differences in major behavioral categories including overall feeding/drinking/movement, time budget, active and inactive state structure, intake and movement bout structure, within-bout structure, and periodicity. These results are provided as Additional file 1: Table 1. For significant behaviors identified by this analysis, we quantified genotypic differences in mouse behavior by one-way ANOVA with genotype as primary factor. In these analyses, multiple comparisons were addressed by Bonferroni correction.

### Periodicity analysis

We examined circadian periodicities using Lomb-Scargle analysis, which detects multiple periodicities within a time series [45, 46]. A major advantage of this approach is that it remains robust in the setting of incompletely sampled data streams. Feeding, drinking, and movement data were binned into 6-min epochs, and significant periodicities (up to 60 h duration) were calculated using an implementation described by [47] and coded in MATLAB 2011b (MathWorks, Natick MA).

## Results

### FDR analysis suggests that *Lgals3*^−/−^ mice demonstrate significant movement deficits

In 2–3 months old mice at α = 0.10, we identified 20 behaviors that significantly differed between the WT and *Lgals3*^−/−^ cohort, 16 of which related to movement (Additional file 1: Table 1, first tab, column 29). By χ^2^ test, movement-related behaviors were overrepresented within this set (16 observed, 7 expected, *p *< 0.009 with critical *p *< 0.016). Similarly, in 6–7 months old mice at α = 0.05, we identified 82 behaviors that significantly differed between the WT and *Lgals3*^−/−^ cohort, 62 of which were related to movement (Additional file 1: Table 1, second tab, column 29). By χ^2^ test, movement-related behaviors were again highly overrepresented within this set (62 observed, 29 expected, *p *< 1.0 × 10^−6^ with critical *p *< 0.016). These results suggest that *Lgals3*^−/−^ mice have a deficit in motor function that progresses with age.

### Movement deficits in *Lgals3*^−/−^ mice

**Fig. 1:**
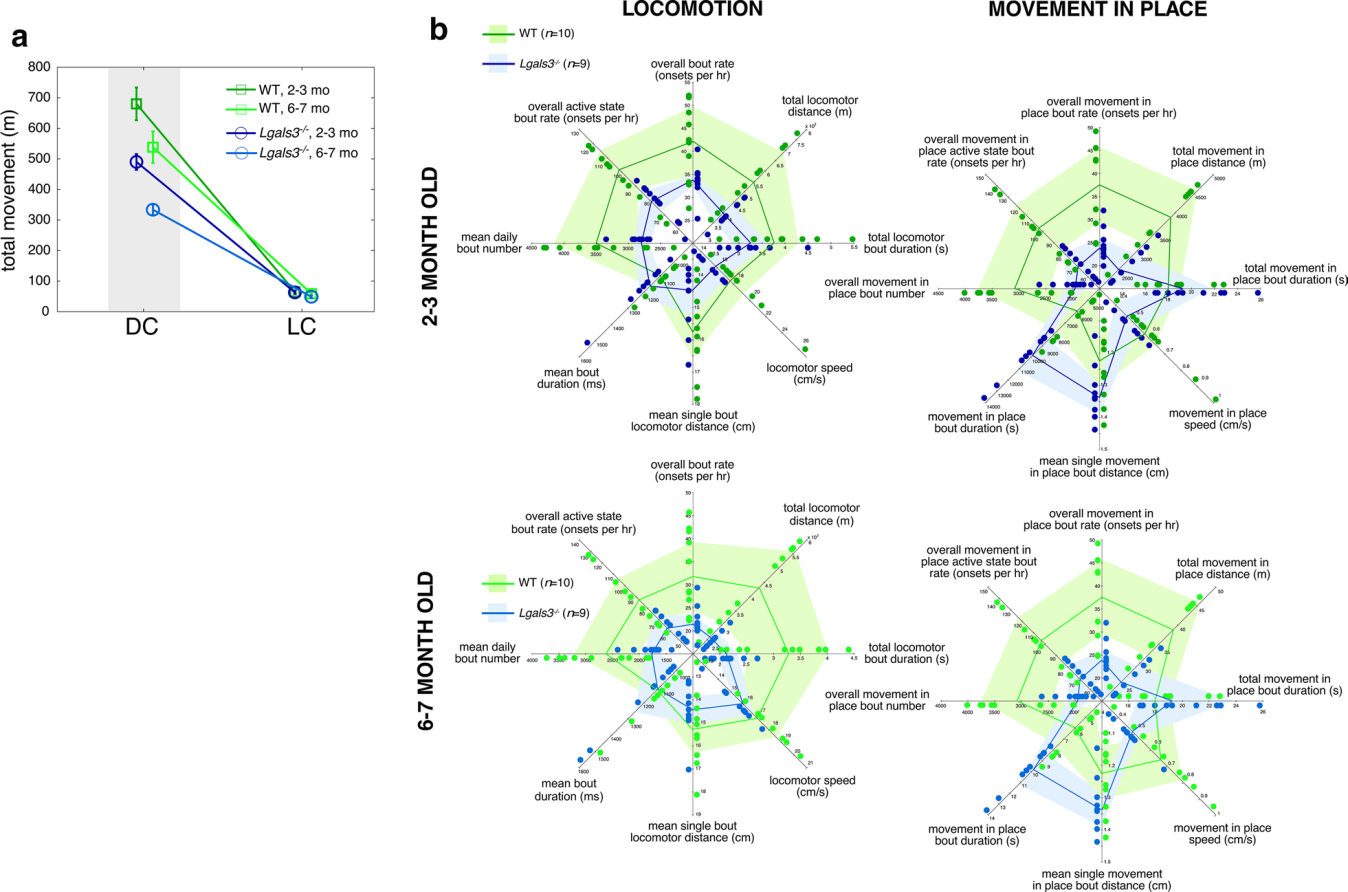
Decreased movement in *Lgals3*^*−/−*^ mice. **a** Overall movement (both locomotion and movement-in-place) averaged by day and by circadian dark/light cycle for 2–3 months (darker traces) and 6–7 months (lighter traces) mouse cohorts. WT mice in green, *Lgals3*^−/−^ mice in blue. Bars are ± one standard error of the mean. Grey background depicts dark cycle. **b** Movement properties. For each polygon graph, all eight axes depict a specific movement property. From the far-right axis moving counterclockwise, values depicted are for total bout duration, total bout distance, bout rate, active state bout rate, bout number, single bout duration, single bout distance, and bout speed. Values for WT mice depicted in green lines with surrounding 95% confidence intervals in light green; individual values for each mouse provided as green circles displaced from the axis. Values for *Lgals3*^−/−^ mice depicted in blue lines with surrounding 95% confidence intervals in light blue; individual values for each mouse provided as blue circles displaced from the axis. Top left: polygon plot for 2–3 months old locomotor bout properties. Top right: polygon plot for 2–3 months old movement-in-place bout properties. Bottom left: polygon plot for 6–7 months old locomotor bout properties. Bottom right: polygon plot for 6–7 months old movement-in-place bout properties

As is evident in Fig. 1a, both 2–3 months old and 6–7 months old *Lgals3*^−/−^ mice demonstrate significantly less total movement compared to WT mice. This phenotype is particularly prominent during the circadian dark cycle, when mice are most active. In an effort to determine the underlying cause of this decreased movement, we subdivide movement into locomotion (consisting of movements performed at high gait speeds with small turning angles) and movement-in-place (consisting of movements performed at low gait speeds with large turning angles). For 2–3 months old mice, we note no significant genotypic differences in any locomotor bout properties (Fig. 1b, top left). However, for 2–3 months old mice we note significant genotypic differences in movement-in-place bout properties of total movement, bout rate, active state bout rate, bout number, and bout duration (Fig. 1b, top right, also see Table 1). In 2–3 months old *Lgals3*^−/−^ mice, movement-in-place bouts were also statistically more likely to be the first behavior performed within new dark cycle active states (WT probability 0.09 ± 0.01; *Lgals3*^−/−^ probability 0.13 ± 0.01, *p *< 0.007 with critical *p *< 0.0125).

**Table 1:** Movement bout properties, 2–3 months old cohort

Behavior	WT (mean ± SD)	*Lgals3*^−/−^ (mean ± SD)	*p*
Movement in place total distance (m)	43.8 ± 7.6	26.9 ± 3.7	< 0.02
Movement in place bout rate (onsets/h)	45.0 ± 9.1	24.7 ± 3.8	< 0.02
Movement in place bout active rate	106.0 ± 20.2	68.6 ± 11.0	< 0.01
Movement in place total number bouts	3736 ± 758	2031 ± 308	< 0.02
Movement in place mean bout duration (s)	6.5 ± 1.7	10.1 ± 1.4	< 0.01

The above movement phenotype was more prominent in 6–7 months old mice. We noted genotypic differences in locomotor bout total distance, overall bout rate, active state bout rate, bout number, and bout duration (Fig. 1b bottom left, also Table 2). These differences led to a statistically significant decrease in the percent time devoted to locomotion within a 24 h time budget (3.8 ± 1.0% WT, 2.5 ± 0.4% *Lgals3*^−/−^, *p *< 0.002 with critical *p *< 0.01). Older *Lgals3*^−/−^ mice demonstrated changes in movement-in-place bouts similar to those observed in younger mice, with genotypic differences in bout total distance, bout rate, bout active state rate, bout number, and per-bout duration (Fig. 1b bottom right, also Table 2). Finally, we again noticed that movement-in-place bouts were statistically overrepresented as the first behavior performed within newly started dark cycle active states (WT probability 0.11 ± 0.01; *Lgals3*^−/−^ probability 0.18 ± 0.01, *p *< 0.004 with critical *p *< 0.0125).

**Table 2:** Movement bout properties, 6–7 months old cohort

Behavior	WT (mean ± SD)	*Lgals3*^−/−^ (mean ± SD)	*p*
Locomotor total distance (m)	422.9 ± 135.5	261.5 ± 29.3	< 0.003
Locomotor bout rate (onsets/h)	33.2 ± 8.6	22.2 ± 3.5	< 0.002
Locomotor bout active state rate	90.5 ± 23.9	61.5 ± 11.0	< 0.004
Locomotor total number bouts	2726 ± 704	1821 ± 289.6	< 0.002
Locomotor total bout duration (s)	3123.5 ± 833.3	2123.4 ± 306.1	< 0.004
Movement in place total distance (m)	37.3 ± 8.5	26.9 ± 4.5	< 0.004
Movement in place bout rate (onsets/h)	37.0 ± 9.2	24.7 ± 3.6	< 0.002
Movement in place bout active rate	100.5 ± 24.9	68.6 ± 11.8	< 0.003
Movement in place total number bouts	3032 ± 759	2031 ± 298	< 0.002
Movement in place mean bout duration (s)	6.4 ± 1.8	10.1 ± 2.0	< 0.0006

### *Lgals3*^−/−^ mice have greater day-to-day heterogeneity in circadian rhythms for movement, food, and water intake

**Fig. 2:**
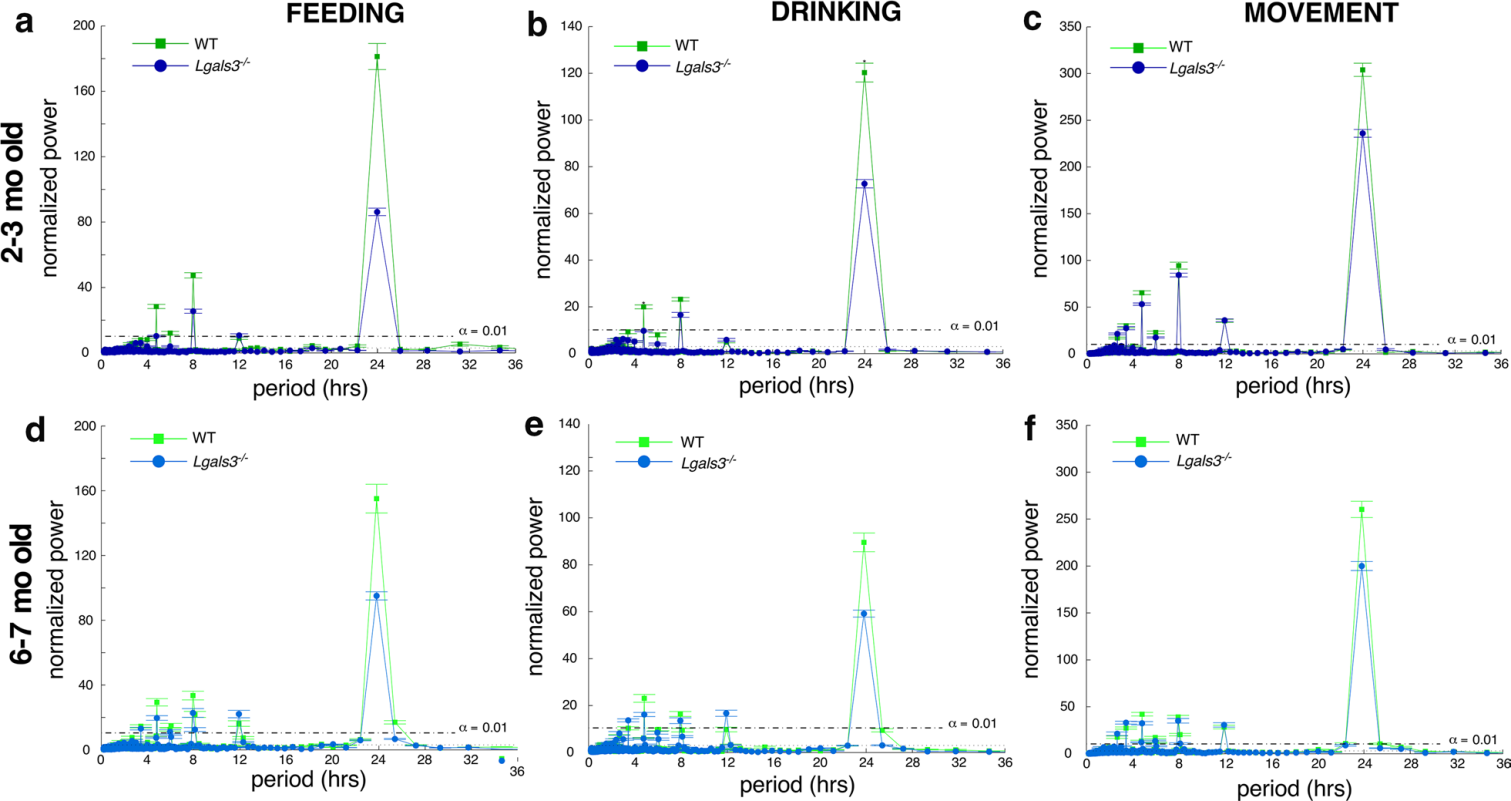
Altered circadian rhythms for feeding, drinking, and movement in *Lgals3*^*−/−*^ mice. Lomb-Scargle periodicity plots depicting significant periodicities (in hours) on x axis, normalized power of specific behavior on y axis. Green shaded lines depict WT responses, blue shaded lines depict *Lgals3*^−/−^ responses. Error bars depict ± one standard deviation of the mean. The dashed line parallel to the x axis depicts the threshold for significant periodicities at α = 0.01. **a** Feeding, 2–3 months cohort. **b** Drinking, 2–3 months cohort. **c** Movement, 2–3 months cohort. **d** Feeding, 6–7 months cohort. **e** Drinking, 6–7 months cohort. **f** Movement, 6–7 months cohort

In both 2–3 months and 6–7 months old mouse cohorts, we note significant decreases in normalized power of the 24-h spectral components for feeding, drinking, and movement behaviors in *Lgals3*^−/−^ mice (Fig. 2). These decreases suggest an increase in the variability of feeding, drinking, and movement over 6 min time windows extending through the duration of our 16 day long observation. We did not appreciate any significant advance or retreat of the daily activity onset and offset times, nor did we find any statistically significant difference in overall active phase duration.

### 6–7 months old *Lgals3*^−/−^ mice have greater body weights than WT cohorts

**Fig. 3:**
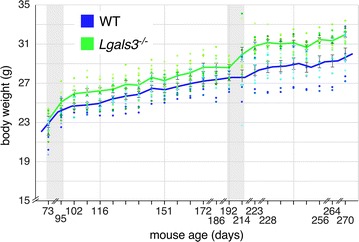
Mouse body mass versus time. Lines depict mean body weights for WT (blue) and *Lgals3*^−*/*−^ (green) mice; error bars are ± one standard error of the mean. Scattergrams for individual mice are depicted by small filled circles. Circles in shades of blue correspond to WT mice; circles in shades of green correspond to *Lgals3*^−*/*−^ mice. Grey bands depict periods where mouse cohorts were tested in the home cage monitoring system. Note that neither axis begins at 0. Sampling interval for x-axis is 7 days except where noted by breakpoints

We found no genotypic differences in neither body weight nor body mass composition between 2 and 3 months old WT and *Lgals3*^−/−^ mice. Similarly, we found no genotypic differences in basal metabolic or activity-associated metabolic rates between 2 and 3 months old WT and *Lgals3*^−/−^ mice. However, 6–7 months old *Lgals3*^−/−^ mice were heavier than their WT counterparts (27.0 ± 1.2 g WT, 28.4 ± 1.3 g *Lgals3*^−/−^, *p *< 0.02). Body mass over the study time period is shown in Fig. 3, and suggests that the two cohorts diverge around 6–7 months of age. Body mass composition parameters noted with this weight change included greater adiposity (14.1 ± 2.1% WT, 16.1 ± 2.0% *Lgals3*^−/−^, *p *< 0.03), greater total tissue mass (23.8 ± 1.0 g WT, 25.3 ± 1.3 g *Lgals3*^−/−^, *p *< 0.01), and greater bone mineral density (0.051 ± 0.003 OD/cm^2^ WT, 0.0054 ± 0.002 OD/cm^2^ *Lgals3*^−/−^, *p *< 0.008) in 6–7 months old *Lgals3*^−/−^ compared to WT mice. No differences in either basal or activity-associated metabolic rates were appreciated between 6 and 7 months old WT and *Lgals3*^−/−^ mice.

### Phenotypes with no difference between WT and *Lgals3*^−/−^ cohorts in both 2–3 and 6–7 months old mice

Regarding home cage phenotypes, we noted no significant differences in overall food or water ingestion between WT and *Lgals3*^−/−^ mice. Except for the previously-noted difference in time allocated to locomotion seen across the 6–7 month old cohorts, there were no significant differences in the 24 h time budgets for feeding, drinking, locomotion, movement-in-place, and resting. There were also no differences in the percentage of time within an active state devoted to feeding, drinking, locomotion, and movement-in-place. We noted no significant differences (overall, or in either circadian dark or light cycles) in active or inactive state properties, including state onset rates, state durations, state transition probabilities, and total numbers of states. Finally, we noted no differences in bout properties of feeding and drinking, including the circadian patterns of total bout intake, bout onset, bout probability, bout duration, bout intensity, and per-bout intake.

## Discussion

There are currently few published studies examining the behavioral consequences of *Lgals3* loss. The Consortium for Functional Glycomics (CFG) spearheaded an impressive effort to obtain baseline behavioral profiles for a wide variety of genes involved in carbohydrate biology, including *Lgals3*. Their results suggested that *Lgals3*^−/−^ mice had diminished freezing in a contextual fear assay, and were more aggressive both in response to an approaching object and in a paired social encounter [48, 49]. *Lgals3* has also been implicated in regulation of energy balance status and development of obesity [50]. Loss of *Lgals3* function was associated with accelerated development of obesity, increased adiposity, insulin resistance, metabolic syndrome, and type II diabetes in mice receiving a high fat diet ([51, 52]; however, data from [53, 54] suggest low *Lgals3* expression as potentially protective against type II DM). *Lgals3* also has been shown to regulate adipose tissue development and function [55–57], and may be an important molecule mediating how diet influences hepatic steatosis [58, 59]. Finally, impaired cognition in older adults has been associated with specific *Lgals3* polymorphisms (rs4644, rs4652, and rs1009977; [60]).

Comparing the above studies to our work, we first note that the CFG investigators observed no genotypic differences in body weight, dark and light cycle metabolic rates, and locomotion. Similarly, we did not find any genotypic differences in food consumption or basal/activity-associated metabolic rates in either 2–3 months old or 6–7 months old mouse cohorts. Values for ${\dot{\text{V}}}$O_2_ reported by CFG for both light and dark cycle epochs were consistent with our measured basal and activity-associated ${\dot{\text{V}}}$O_2_. We also noted no genotypic differences in body weight between 2 and 3 months WT and *Lgals3*^−/−^ mice. However, 6–7 months old mice (slightly older than the oldest reported in [48]) had increased body weight and greater adiposity in *Lgals3*^−/−^ compared to WT cohorts. Since the Teklad 7012 diet is 17% fat, and mouse dietary requirements are estimated at 5% fat [61], it is reasonable to suggest that the increase in *Lgals3*^−/−^ body weight/adiposity we observe in part replicates prior findings of high-fat-diet-induced obesity.

We provide the first data suggesting *Lgals3* involvement in motor system development and/or performance. Specifically, we noted an overall ~ 30% decrease in *Lgals3*^−/−^ movement (both locomotion and movement in place) compared to WT mice. This decrease was observed in an acclimated home cage over 16 days, and thus does not assess the same construct reported by the CFG investigators, who found no change in open field locomotion over 30 min. *Lgals3*^−/−^ mice therefore display decreased home cage locomotion with no change in novelty-evoked locomotion. The moderate increase in *Lgals3*^−/−^ body weight may slightly increase the behavioral cost of movement, and thus decrease total movement. However, the prominent differences in movement-in-place bout rate, active state bout rate, bout duration, and bout number observed in 2–3 months old mice, as well as the large differences in both locomotion and movement-in-place bout rate, active state bout rate, bout duration, and bout number observed in 6–7 months old mice, suggest that *Lgals3* constitutive loss evokes functional deficits in underlying motor substrates. Both gene (ebi.ac.uk/gxa, informatics.jax.org/expression.shtml) and protein expression (emouseatlas.org, proteinatlas.org) atlases suggest that *Lgals3* expression (at low-to-moderate levels) occurs in both pre-and post-natal brain, and has been localized to regions involved in motor behavior generation, including the cortex, striatum, cerebellum, and spinal cord. We thus argue that *Lgals3* loss alters mouse motor function, either through its impact on motor development or through altered neuronal signaling in CNS regions that regulate or produce motor behavior. Further studies examining the consequences of *Lgals3* loss at synaptic, neuronal, ensemble, and tissue levels of organization will be required to determine the precise mechanisms underlying this functional loss.

As mentioned earlier, *Lgals3* has been implicated in a large number of physiological tasks at both a cellular and organwide level of organization. It is thus notable that mice with complete loss of *Lgals3* function demonstrate relatively few behavioral differences when compared to wildtype C57BL/6J mice. This finding suggests that, at least in the mouse, there is some genetic redundancy regarding *Lgals3* function. Studies of galectin evolution focusing on intron/exon organization as well as sequence identity suggest that duplication of ancestral galectin genes in animal lineages preceding the first teleost fish [62] provided the precursors for what has become a large vertebrate protein family [63]. There is also data suggesting that galectins may be able to substitute for one another in specific circumstances. For example, Lgals1 may compensate for *Lgals3* loss at the spliceosome [64]. Extracellular Lgals1 also regulates T cell apoptosis in a manner similar to that of extracellular *Lgals3* [65]. The behavioral phenotype arising from *Lgals3* functional loss thus identifies neuronal loci and processes where there is no compensation for gene loss.

Finally, these findings support the hypothesis that loss of molecules with specific pattern recognition properties (in this case, for β-galactosidase linkages) evokes behavioral phenotypes potentially arising from inappropriate neuronal synaptogenesis, pruning, and/or maintenance. There is already clear evidence that molecules able to recognize specific protein regions play crucial roles during CNS development [66, 67]. This study further implies that molecules able to recognize specific carbohydrate regions may have parallel roles during CNS development. Further efforts to understand carbohydrate recognition in the developing brain are thus clearly justified, and may provide important and clinically relevant insights into significant psychiatric conditions, including autism spectrum disorders [68] and schizophrenia [69].

## Conclusions

This study provides the first data describing home cage feeding, drinking, movement, and circadian rhythm in mice cohorts constitutively lacking *Lgals3* function. We performed a longitudinal assay of these behaviors at 2–3 and 6–7 months of age. At both ages, *Lgals3*^−/−^ mice showed less home cage movement compared to WT. This decrease was due to decreases in both forward locomotion and movement-in-place. These differences grew more pronounced with age. In older mice, we could further determine that decreased movement was a result of lower bout initiation rates (for both locomotor and movement-in-place bouts), with similar distances traversed per bout. Lower bout initiation rates also led to lower total numbers of locomotion and movement-in place bouts. *Lgals3*^−/−^ mice at both ages also had more heterogeneous circadian patterns of feeding, drinking, and movement compared to WT mice.

## Additional file

**Additional file 1: Table 1.** *Lgals3*^−/−^ and WT behavioral metrics. Column 1 lists the abbreviation for each behavior, Column 2 lists the overall behavioral assay class, Column 3 provides a brief description of the behavior, Column 4 is an index. Columns 5 and Column 6 list mean values for each behavior, control and *Lgals3*^−/−^ respectively. Columns 7–25 list results for each test by individual mouse. Control mice are listed in columns 7–16; *Lgals3*^−/−^ mice are listed in columns 17–25. Columns 26, 28, and 30 provide unadjusted *p* values calculated by Mann–Whitney, Student’s *t* test, and fold change (B) methods. Columns 27 and 29 provide *p* values adjusted by false discovery rate (FDR) for Mann–Whitney (column 27) and Student’s *t* test (column 29). Column 31 lists the behavioral fold change. Labeled spreadsheet tabs correspond to 2–3 months old and 6–7 month old cohorts, respectively.

## Abbreviations

*Lgals3*: galectin-3
CRD: carbohydrate recognition domain
DEXA: dual energy X-ray absorptiometry
BMD: bone mass density
BMC: bone mineral content
BArea: bone area
TArea: tissue area
R_ST_: ratio of soft tissue attenuation
TTM: total tissue mass
${\dot{\text{V}}}$O_2_: maximum oxygen uptake
DO_2_: global oxygen delivery
O_2_out: oxygen output
${\dot{\text{V}}}$CO_2_: maximum CO_2_ production
DCO_2_: global CO_2_ removal
CO_2_out: CO_2_ output
FDR: false discovery rate
WT: wildtype
